# Integration of multiscale dendritic spine structure and function data into systems biology models

**DOI:** 10.3389/fnana.2014.00130

**Published:** 2014-11-12

**Authors:** James J. Mancuso, Jie Cheng, Zheng Yin, Jared C. Gilliam, Xiaofeng Xia, Xuping Li, Stephen T. C. Wong

**Affiliations:** ^1^Department of Systems Medicine and Bioengineering, Houston Methodist Research InstituteHouston, TX, USA; ^2^TT and WF Chao Center for Bioinformatics Research and Imaging for Neurosciences, Houston Methodist Research InstituteHouston, TX, USA

**Keywords:** dendritic spines, microscopy, image analysis, systems biology, modeling and simulations, big data

## Abstract

Comprising 10^11^ neurons with 10^14^ synaptic connections the human brain is the ultimate systems biology puzzle. An increasing body of evidence highlights the observation that changes in brain function, both normal and pathological, consistently correlate with dynamic changes in neuronal anatomy. Anatomical changes occur on a full range of scales from the trafficking of individual proteins, to alterations in synaptic morphology both individually and on a systems level, to reductions in long distance connectivity and brain volume. The major sites of contact for synapsing neurons are dendritic spines, which provide an excellent metric for the number and strength of signaling connections between elements of functional neuronal circuits. A comprehensive model of anatomical changes and their functional consequences would be a holy grail for the field of systems neuroscience but its realization appears far on the horizon. Various imaging technologies have advanced to allow for multi-scale visualization of brain plasticity and pathology, but computational analysis of the big data sets involved forms the bottleneck toward the creation of multiscale models of brain structure and function. While a full accounting of techniques and progress toward a comprehensive model of brain anatomy and function is beyond the scope of this or any other single paper, this review serves to highlight the opportunities for analysis of neuronal spine anatomy and function provided by new imaging technologies and the high-throughput application of older technologies while surveying the strengths and weaknesses of currently available computational analytical tools and room for future improvement.

## Systems biology

**Figure 1:**
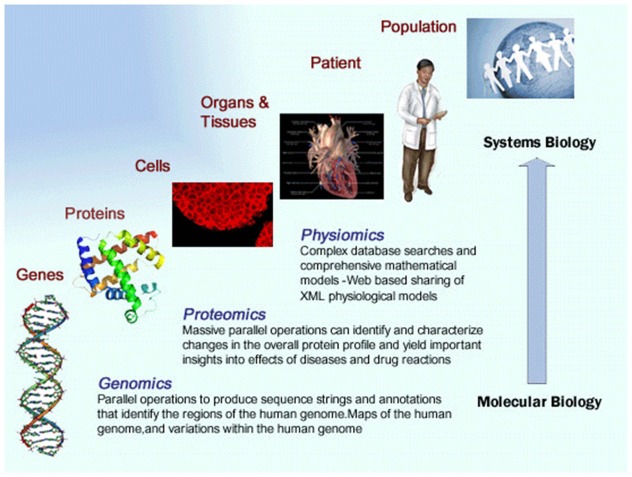
**Illustrates the principles of the systems medicine approach**. Multi-scale data from a range of sources is integrated to provide a more comprehensive understanding of a disease state.

The nervous system is so complex that studies have had to focus on individual or small numbers of components at a single level of organization. Such a reductionist approach has helped reveal many processes that govern neuronal and brain function and has led the foundation of the field of neuroscience. Translating the logic of this reductionist approach to modern neuroscience results in a framework that is rooted in the scientific method and premised on biological discovery and understanding of disease mechanisms. Although the logic remains compelling, the traditional reductionist framework often leads to the study of individual proteins, subcellular structures, or neurons in isolation and can neglect the dynamic interaction of all components and how their interaction can lead to a more comprehensive understanding of neuronal function and dysfunction. Owing to recent advances in biotechnologies and computational sciences, biologists are now gaining the capability to go beyond the interactions of components within a single level of biological organization and the study of one or a few components at a time. The application of engineering precepts to biological systems has spawned the field of systems biology. Biologists are increasingly able to integrate information and form networks of interacting components across many organisms, from multiple levels of biological organization, such as cells, organs, and populations, and about entire systems, such as all the genes in a genome, to obtain new knowledge that incorporates more of the complexity that characterizes biological systems (see Figure 1).

Recent advances in imaging approaches have provided investigators with the means to acquire not only quantitative information about fine neuronal structures, including dendritic spines, *in vivo* or *in vitro* but also to examine their complete biological context including interactions with neighboring cells, connectivity, and chemical and protein composition. To date, comprehensive approaches to integrate these large data sets from fluorescence microscopy, electron microscopy (EM), and various imaging modalities, such as coherent anti-Stokes Raman spectroscopy (CARS), second harmonic generation (SHG), and autofluorescence imaging, that take advantage of intrinsic signals in living biological samples have been slowed by signal to noise issues and the lack of automated analysis algorithms. This has hindered their full use in a comprehensive systems biology analysis of dendritic spine pathology, and relegated their use to experimental paradigms that address biomedical questions from a traditional, reductionist standpoint.

**Figure 2:**
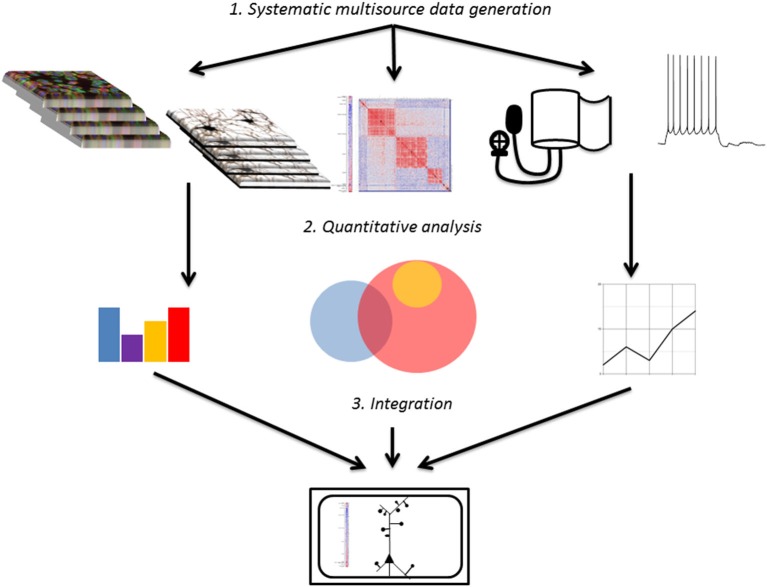
**Overall workflow schematic**. Systematic raw data sets from diverse sources are collected (or extracted from public databases) and analyzed to provide manageable input for modeling. Data sets are then integrated to create models of function and disease that can be used to predict physiology and therapy.

The principle steps guiding the development of systems medicine models from brain imaging data are as follows: (1) generation and collection of large volumes of relevant imaging data; (2) systematic analysis and quantification of imaging data; (3) integration of analyzed imaging data with other data sources to create a systems level model of disease. This review primarily focuses on progress made to date on the first two steps as well as room for future improvement, while touching briefly on the promise of the third step (Figure 2).

## Neuronal anatomy (including dendritic spines)

Elements of the central nervous system are well known to exhibit strong correlations between anatomy and function on a number of different scales (Lee et al., 2012; Wang et al., 2013). Various subpopulations of neurons show variations in morphology which often underlie fundamental differences in signal integration and transmission properties of the individual subpopulations; the integration of these many diverse neuronal subtypes forms the foundation for basic and higher order brain processes. Throughout the nervous system various processes, both normal, such as development and aging, and pathological, such as neurodegeneration and drug addiction, manifest as changes in neuronal anatomy (Elston and Rosa, 1997, 1998; Engert and Bonhoeffer, 1999; Maletic-Savatic et al., 1999; Elston, 2000; Jacobs et al., 2001; Elston et al., 2011a; Bosch and Hayashi, 2012). While the study of neuronal anatomy alone may not be sufficient to ascertain a full understanding of neuronal circuit and brain disease pathology, the large data sets that are readily obtained from high resolution systematic imaging of brain regions provide an excellent resource for integration into comprehensive, big data models of neuronal function and pathology (Jacobs et al., 1997; Elston et al., 2009, 2010a,b, 2011b; Sasaki et al., 2014). Full understanding is far off due to the sheer complexity of the nervous system; there are approximately 10^11^ neurons making approximately 10^14^ synaptic connections (Williams and Herrup, 1988; Nimchinsky et al., 2004). A comprehensive evaluation of neuronal morphology and connectivity far exceeds the limits of our abilities to gather and analyze data at this time.

Among the most notable and plastic neuronal structures, dendritic spines are post-synaptic protuberances found primarily at non-symmetrical excitatory synapses directly opposed to a presynaptic bouton as illustrated by the high resolution of EM (Jones, 1968). Normally composed of a round spine head with a volume ranging from 0.015 to 0.77 µm^3^ (DeFelipe et al., 1988; Petralia et al., 1994a,b,c; Knott et al., 2006; Arellano et al., 2007) and a thinner spine neck, dendritic spines serve as the point of contact between two neurons highly enriched in postsynaptic signaling components such as GluR (De Paola et al., 2006). They provide isolated sites for local integration and molecular compartmentalization of second messenger pathways such as calcium essential for normal synaptic scaling and learning and memory (Shepherd, 1996; Yuste et al., 2000; Yuste and Bonhoeffer, 2001; Alvarez and Sabatini, 2007). A rich literature exists of comparative studies among brain regions and species, allowing us to discover correlations between brain and neuronal anatomy, particularly in dendritic spines, and cognitive differences in healthy animals (Benavides-Piccione et al., 2002; Ballesteros-Yáñez et al., 2006).

## Dendritic spine abnormalities and cognitive impairment

It has long been recognized that neurodegenerative disease and other neurological pathology often manifests as an alteration in neuronal anatomy and in particular in the number, shape, and distribution of dendritic spines (Goldman-Rakic, 1996; DeFelipe, 2004; Alonso-Nanclares et al., 2005; Glausier and Lewis, 2013; He and Portera-Cailliau, 2013; Licznerski and Duman, 2013; Pozueta et al., 2013; Smith and Villalba, 2013; Villalba and Smith, 2013; Wong and Guo, 2013), most likely correlating with alterations in neuronal signaling and axonal and neuronal death. Additionally, treatments that reduce the cognitive symptoms of neurodegenerative disease also reverse spine pathology (Smith et al., 2009). Unfortunately for patients and clinicians, these alterations can only be observed in small amount of tissues sampled from specific brain areas via biopsy, or post-mortem tissue received from deceased patients. The accumulation of senile plaques and tau bundles are the most well-known anatomical hallmarks of Alzheimer’s disease, but it is synapse loss, exemplified by a reduction in dendritic spine density in the cerebral cortex and hippocampus, that best correlates with disease progression (Moolman et al., 2004). A decrease in length and complexity of the dendritic arbor as well as a significant reduction in spine density in medium spiny neurons of the dopamine receiving areas of the brain have long been observed as pathological hallmarks of Parkinson’s disease (Stephens et al., 2005). Rett syndrome patients display a prominent reduction in dendritic arbor complexity, dendritic spine density, total number of neurons, and total brain volume which appears within the first year of the patient’s life (Belichenko and Dahlström, 1995a,b; Armstrong, 2005). That these anatomical abnormalities are recapitulated in animal models (Smrt et al., 2007; Belichenko et al., 2009) of the disease reveals that Rett Syndrome is a disorder of neuronal development and reconfirms the correlation between dendritic spine anatomy and neuronal function or dysfunction.

Epilepsy is a neurological disorder resulting from network hyperactivity in neuronal circuits that causes chronic seizures (Bromfield et al., 2006). The principle hyperactive neurons in epilepsy are found in the medial temporal lobe and are characterized by dense excitatory synaptic inputs through dendritic spines. As one might expect, the chronic hyperactivity found in epilepsy correlates with significant changes in spine density, but it is unclear whether spine loss is a cause of epilepsy or a compensatory change in response to it (Wong and Guo, 2013). Further complicating the issue anatomical, and functional changes in the brain coincide with gene transcriptional chances, but a cause on effect relationship has not been established in either direction (Arion et al., 2006). This situation is not the exception but the rule for post mortem studies of diseased human tissue.

Here animal models of human disease are extremely useful for elucidating the causes and mechanisms of synaptic dysfunction. Not only do they offer the lack of confounding genetic and environmental factors found in human patients, but they offer investigators the potential to study disease progression at defined time points. Still, the use of imperfect animal models of human disease yields an understanding of these diseases and disease mechanisms that is overly simplistic (Chesselet and Carmichael, 2012) and the validity of these models in accurately predicting human disease is highly variable. Only comprehensive integration and iteration of clinical studies and mechanistic studies in animal models (under constant evaluation) can provide an accurate vision of the causes and mechanisms of complex neurodegenerative diseases.

Directed anatomical studies of neurons and populations of neurons along with protein expression and electrophysiological studies have been instrumental in the development of models that predict how the electrical properties of neurons vary with changes in cell shape; these works have contributed to a large number of popular software packages that model neuronal activity under various conditions (Hines and Carnevale, 2001; Bower and Beeman, 2007). The field of systems and computational neuroscience is mature and continues to flourish. In addition, a wide range of complementary knowledge can be gained by systematic analysis of available data sets and the acquisition of new high throughput data sets; chief among these are image data sets on multiple scales and from a variety of modalities which to data remain largely underutilized.

## Multiscale imaging approaches (from big to small)

Clinical imaging approaches such as CT, PET, and MRI have successfully illustrated pathological hallmarks of neurodegenerative disease such as loss of brain mass (Thompson et al., 2007; Schuff et al., 2009), alterations in long range connectivity (Daianu et al., 2013), and toxic protein accumulations such a senile plaques (Cohen et al., 2012; Johnson et al., 2012). These approaches, fMRI in particular, provide functional information regarding systems-level neuronal signaling and pathology, but lack the resolution for imaging individual synapses or subcellular components such as dendritic spines. Still, they provide another valuable resource for integration into systems biology models of brain function and pathology and excellent work, including the Alzheimer’s Disease Neuroimaging Initiative (ADNI) and related projects (Mueller et al., 2005; Bradshaw et al., 2013; Daianu et al., 2013) is already taking advantage of it. A full account of the uses of these clinical imaging approaches or their limitations is beyond the scope of this review.

**Figure 3:**
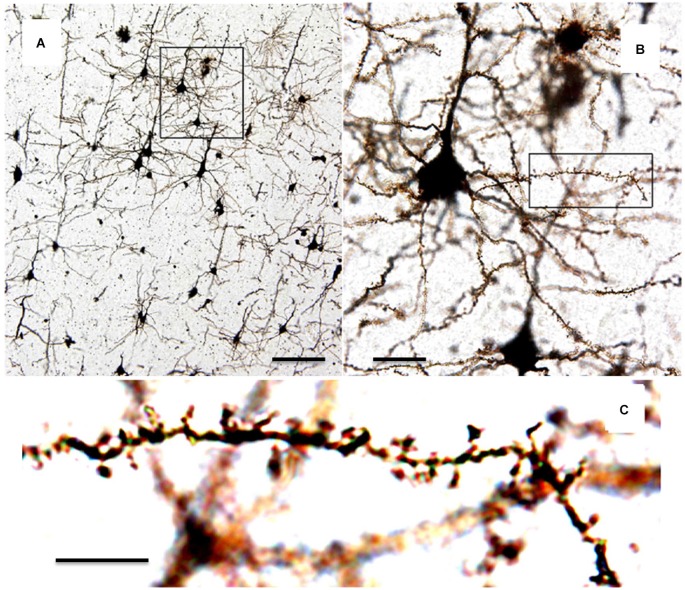
**Photomicrographs of Golgi-stained mouse cortical neurons from slices**. **(A)** Neurons in brain slices are randomly and sporadically labeled allowing visualization of individual neurons. Scale = 50 µm. **(B)** Enlargement of boxed area in **(A)** showing dendrites and spines belonging to individual neurons. Scale = 10 µm. **(C)** Enlargement of boxed area in **(B)** showing individual dendritic spines which can be counted and analyzed. Scale = 5 µm. (adapted from Mancuso et al., 2013).

Determination of the number, morphology, and dynamics of synapses in populations of neurons is a well-accepted metric for neuronal health, development, and function that can provide investigators with information about specific brain regions and neuronal subtypes. To date, optical microscopy provides one of the most attractive tools for mechanistic studies of synaptic health and dysfunction applicable to both neurodegenerative disease patients (post-mortem tissues) and animal models of human disease. Dating back over 100 years, Golgi staining and brightfield microscopy of brain tissue sections has long been the workhorse for studies of neuronal anatomy. Invented by Camillo Golgi in his kitchen, the Golgi stain is elegant in its simplicity. It is based on the precipitation of silver or mercury chromate granules along the membranes of neurons in fixed tissue. These dense black particles clearly and stably stain neurons, leaving their anatomy including fine structures visible to bright field microscopy with high signal to noise (Pannese, 1999). It is particularly worth noting that the Golgi method randomly and sparsely labels cells throughout a field of view, allowing differentiation of the fine processes of a cell of interest from the dense background of connected circuitry (Figure 3). Santiago Ramon y Cajal took advantage of this fantastic approach to painstakingly document the anatomy of cells throughout the nervous system. For this combination the two men shared the Nobel Prize in Physiology or Medicine in 1906 (Grant, 2007).

Since that time these approaches have been widely modified and modernized and cell filling and stereology have been the workhorses in gathering data that have led to a systematic understanding of neuronal structure down to the synapse level (Elston, 2003; Elston and Fujita, 2014). Standard tissue preparation, staining, and bright field microscopy, while an extremely effective approach in the identification, characterization, and quantification of dendritic spines from specific regions of the brain, does have its limitations. Principle among these is the fact that tissues used in these approaches are by their very nature taken post-mortem and processed. Additionally, because bright field microscopy achieves no separation between photons originating at the focal plane and those arising above or below, 3-dimensional resolution is lacking; a considerable amount of neuronal structure is lost, simply because the nervous system does not exist entirely in two dimensions. For instance, the number of dendritic spines as measured by brightfield microscopy is greatly underestimated due to the exclusion spines that only protrude from the parent dendrite in the z-plane (Harris and Stevens, 1988). Slices also must be cut extremely thin, which eliminates most long range synaptic connectivity. Finally, the two-dimensional resolution limits observation of changes in structures below the micron range, which is appropriate for changes in dendritic spines but nothing smaller including structures in the spine such as post-synaptic density.

## *Ex vivo* and *in vivo* imaging

All fixed tissue imaging approaches by design face that limitation that they provide only a snapshot of neuronal anatomy at a given time. Only imaging of live cells, tissues, or organisms allow for the observation of dynamic processes. The widespread genetic introduction of fluorescent indicator dyes and recombinant fluorescent proteins derived from jellyfish has revolutionized live cell imaging including the imaging of neurons. Using transgenic animal models or viral transduction, fluorescent proteins such as GFP have been introduced into specific, genetically defined populations of neurons and other neural cells (Feng et al., 2000; Gong et al., 2003). Random insertion of transgenes leads to mouse models with neurons labeled sparsely enough to allow for high resolution imaging of fine anatomical structures such as dendritic spines (Feng et al., 2000). The combination of fluorescent labels with confocal microscopy, which uses a pinhole to discard those photons arising outside of the focal plane, allows these processes to be imaged at near diffraction resolution in three-dimension (3D). Due to their electrical excitability, electrophysiological recording is of course the standard method to observe and manipulate the function of neurons or populations of neurons. Still, confocal microscopy is limited by the scattering of excitation and emission photons to imaging structures 50 µm below the surface due to photodamage that occurs with the higher laser powers necessary with increasing imaging depth.

By reducing light scattering in the excitation path and restricting fluorescence activation to a tiny focal volume, allowing for 100% collection of emission signal, non-linear, multiphoton imaging with near infrared wavelengths, has enabled researchers to image neuronal structures as far as 1 micron deep (Theer et al., 2003) and over extended periods without significant light-induced tissue damage. Live tissue imaging coupled with physiological studies provides the capability to correlate changes in neuronal anatomy with functional measurements and manipulations in the same neuron or neurons (Kasai et al., 2010). Indeed such studies have led to our understanding of the mechanisms of synaptic plasticity at the single synapse level (Zhou et al., 2004; Yang et al., 2008) as well as the entry, diffusion, and intracellular release of calcium caused by synaptic activation and downstream signaling events and the role of dendritic spine morphology (Denk et al., 1995; Yuste et al., 1999; Holthoff et al., 2002; Araya et al., 2014).

The convergence of advances in microscopy and genetic tools for neuronal labeling has led to the advent of high resolution *in vivo* imaging of neuronal function and anatomy including imaging of the dendritic spine dynamics in awake, moving animals (Dombeck et al., 2009; Scott et al., 2013). This allows investigators to look at changes in dendritic spine density and anatomy and how they relate to the response of an animal to normal environmental stimuli (Jung and Herms, 2014). A number of studies have combined anatomical imaging and stereological analysis of neuronal structure including dendritic spines with electrophysiology by filling cells with fluorescent dye through the recording pipette and other imaging modalities in order to paint a picture of the elaborate changes that occur in models of disease (Day et al., 2006).

Formation and pruning of spines as well as changes in spine size and shape are a part of normal development, essential for the function of neuronal circuits (Calabrese, 2006). While more than one mechanism of synapse formation exists, in the most common model of neuronal development a postsynaptic neuron projects numerous small, thin filapodia which receive random inputs from neighboring synaptic terminals (Bhatt et al., 2009). The filipodia that receive sufficient inputs of the proper timing, mature into full dendritic spines and form a specific synapse, while those that do not are eliminated. While in adulthood most dendritic spines are stable over a long period of time, a proportion appear and disappear in any given period according to their inputs and it is thought that these changes highlight experience-dependent remodeling of neuronal circuits (Knott and Holtmaat, 2008). More specifically, it has been demonstrated in adult neurons that changes in the size and shape of dendritic spines correlate with plasticity at individual synapses (Matsuzaki et al., 2004).

All cellular level biological processes and synapse dynamics specifically are extremely dependent on the context of their cell-cell interactions and general environment (Hu and Wang, 2010; Petzold and Murthy, 2011; Van Beek and Claassen, 2011; Dallérac et al., 2013), which is a fundamental facet of systems medicine. It should be noted that the aforementioned fluorescence techniques based on protein expression or dye labeling only show labeled structures and neglect surrounding tissue. Label-free imaging approaches, while exhibiting much lower signal to noise, are then essential for placing labeled structures in the proper biological context. Among these, two photon autofluorescence (TPEAF) imaging, taking advantage of autofluorescent metabolic intermediates such as NADH, has long been used to image cells and distinguish among populations of cells based on metabolic rate (Williams et al., 1994; Piston et al., 1995; Monici, 2005). Second harmonic generation is another valuable non-linear, label-free imaging approach often used to visualize ordered arrays of proteins as are seen in structures such as cytoskeleton (Vanzi et al., 2012). More recently CARS imaging and its various subtypes have emerged as valuable tools for measuring chemical diversity in biological samples with high spatial resolution and is now used to distinguish cells based on the distribution of specific biological moieties, particularly various species of lipid molecules (Evans and Xie, 2008). Recently, the Wong lab developed a novel multimodal imaging approach combining TPAEF, SHG, and CARS in order to differentiate lung cancerous tissue from normal and desmoplastic edge (Xu et al., 2013). Lipid droplets and cell nuclei were well characterized in CARS images, and elastin and collagen fibers were illustrated by TPEAF and SHG modalities, respectively. A major obstacle to using these modalities as a component for systems levels models of neuronal function and dysfunction has been the development of appropriate computational tools for the systematic and quantitative analysis of such multimodal data. In this case the signal to noise issues that plague label-free approaches were improved by employing Sternberg’s rolling ball method (Sternberg, 1983) to adaptively estimate background followed by denoising. Images were then processed using the eigenvalues of a Hessian matrix to find “tube-like” structures and a Robust Automatic Threshold Selection (RATS) to complete the segmentation (Wilkinson and Schut, 1998). With any such multimodal approach no one size fits all image analysis paradigm exists due to heterogeneity in signals and in anatomy, as such, specific approaches are necessary for each.

### Super-resolution and electron microscopy

Spine size, shape, and number alone do not give a complete portrayal of synaptic changes, both normal and pathological. Modulation of normal synapse physiology and function is accompanied by architectural rearrangements of intra-terminal ultrastructures that occur on timescales ranging from milliseconds to minutes. Macromolecular fluctuations of this magnitude are difficult to study using conventional imaging and biochemical methods as these methods lack the blend of resolution, cellular context, stability, and sensitivity needed to study native cellular processes. Neurological diseases are caused by genetic mutations affecting these native processes. Many of these mutations are also suspected of altering the ultrastructural architectures within neurons at the nanometer level.

Traditionally, EM has been employed to examine these nanoscopic subcellular changes (Kuwajima et al., 2013). Originally it was Gray (1959a,b) who used EM to conclusively demonstrate that the dendritic spines of pyramidal neurons are in fact the sites of synaptic contact. The postsynaptic density (PSD), the electron dense region of synaptic contact located on the distal tip of dendritic spines, has been a highly effective target of EM and its anatomy well-characterized in various brain regions (Cohen et al., 2012). In addition the application of proteomics approaches such as matrix-laser desorption/ionization-time-of-flight (MALDI-TOF) mass spectrometry (MS) have enabled researchers to gain a quantitative working knowledge of the protein composition of PSDs from a variety of brain regions in a number of neuronal subtypes under number of different conditions. While these results continue to suffer from some degree of false negatives and positives due to the nature of the detergent solubilization protocols used to isolate PSD proteins (Sheng and Hoogenraad, 2007), further refinement of data generation approaches for PSD proteomics will make data gained an excellent candidate for incorporation into our modeling approach.

Work from the Reid lab (Bock et al., 2011) provides an excellent example of integration of a large scale reconstruction of brain connectivity from EM data with *in vivo* functional data. Using intravital microscopy of a fluorescent calcium indicator in order to elucidate a single visual functional trait, orientation selectivity of a group of neurons, the group then correlated that activity with synaptic connectivity terminating in dendritic spines as determined by EM. It should be noted that the data acquired by this study at the lowest resolution necessary to carry out these goals on a single area of the visual cortex was upwards of 35 TB and characterization of this huge data set was entirely manual and constituted a herculean effort. Systematic application of this type of approach in normal and diseased tissue on a scale necessary to formulate models about function and dysfunction in synaptic circuitry certainly awaits a more rapid, less labor intensive analytical approach.

More recently, superresolution light microscopy approaches have arisen that allow for the examination of dendritic spine processes on a nanometer scale (Bethge et al., 2013; Loew and Hell, 2013; Takasaki et al., 2013). Taking advantage of the intrinsic properties of fluorophores or fluorescent proteins, these approaches, such as STED, STORM, and PALM, circumvent the normal diffraction barrier, allowing for light microscopy at nanometer resolution. Because they do not require special processing of the tissues involved, these approaches are compatible with live cell imaging. The combination of superresolution light microscopy with two photon excitation enabled researchers to resolve structures as small as the dendritic spine neck in thin slices and leaves open the possibility of accomplishing the same task *in vivo*. A recent study from the Rizzoli lab (Wilhelm et al., 2014) provides an excellent example of an approach to incorporate superresolution imaging of the synapse with omics data, in this case proteomic data, in order to construct a comprehensive model of the presynaptic bouton with the potential to expand our understanding of vesicle release and synaptic signaling. While this particular biological preparation, due to its simplicity, lends itself beautifully to such a comprehensive approach, a modified approach could certainly be applied to a number of other more complex preparations.

Cryo-electron tomography (cryo-ET) has emerged as a powerful technique, well-suited to imaging normal and abnormal macromolecular assemblies while preserving their native cellular contexts. Cryo-electron tomography is a 3D imaging technique that is increasingly being utilized to study the close relationship between neuron function and subcellular organization (Harapin et al., 2013; Lučič et al., 2013). The strength of cryo-ET centers on its use of isotonic cellular preservation, which captures fragile cellular structures in their native, near-physiological state. Cryopreserved specimens are imaged on an electron microscope while being tilted from −70° to +70° during the imaging process. The observed cellular features are imaged as a collection of 2D micrographs that are computationally combined into a single 3D tomogram, which is a nanometer-scale map of the original cell (Milne et al., 2013). The high-quality spacial information contained within these tomograms has revealed detailed views of eukaryotic cells that, if applied toward neurological disease, hold the potential to further the understanding of neurodegeneration.

Synapses are comprised of the necessary pre- and post-synaptic biological components for sending, receiving, and responding physiologically to neuromodulatory signals. These components include biosynthetic organelles such as endoplasmic reticula and mitochondria, various lipid-based cellular membranes, membrane delineated ion channels and protein complexes, cytoskeletal assemblies and their associated cellular trafficking machinery, and lipid vesicles bearing chemical neurotransmitters. Changes in one synaptic component, inevitably lead to compensatory changes in other related components. Fluctuations in the presynaptic membrane potential are known to modulate the rate of synaptic vesicle fusion with the plasma membrane and the release of chemical neurotransmitter into the synaptic cleft. Previous cryo-ET studies have shed light on the arrangements of presynaptic cytoskeletal structures (Fernández-Busnadiego et al., 2010), such as actin filaments and microtubules, as well as the number of transmitter-containing vesicles and their distributions in conventional (Fernández-Busnadiego et al., 2013; Harlow et al., 2013) and ribbon-type active zones (Lenzi et al., 1999). Similar methods could conceivably be used to extend the current understanding of vesicle numbers and fusion (Bharat et al., 2014) in diseased neurons as a proxy for measuring neurotransmitter release kinetics in diseases, such as Alzheimer’s or Parkinson’s, known to affect cognitive function. Similarly, cryo-ET could be applied to human diseases to investigate macromolecular structures downstream of transmitter release at the synaptic cleft (Lucić et al., 2005) and postsynaptic nerve terminals located on dendritic spines (Swulius et al., 2012). Because spine density is affected in neural pathologies, there is a clear link to the critical nature of synaptic junctions and dendritic spines in normal neuron function. Currently, spine count is primarily used as an indicator of neural distress, but little is known about the early morphological changes that occur within the synapse that contribute to the numerical decline of dendritic spines. By elucidating ultrastructures on either side of the synapse, depth of understanding could be gained regarding the changes occurring within neurons affected during Alzheimer’s or Parkinson’s, and also whether changes in spine density follows a unified progression or whether each affected spine holds clues to further understanding of disease.

Mitochondria play a critical role in supporting the energy demands of neurons, and dysfunctional mitochondrial metabolism has been implicated in the pathogenesis of a number of neurodegenerative diseases (Itoh et al., 2013; Maresca et al., 2013). Mitochondria are present along the axon length and within pre- and postsynaptic termini. Within or near dendritic spines, mitochondria are thought to generate sufficient energy for ATP-intensive processes including kinesin-based protein and vesicular transport, endocytosis and recycling of neurotransmitter receptors, as well as biosynthetic responses resulting from presynaptic stimuli. In addition, mitochondria are a major source of reactive oxygen species (ROS) within eukaryotic cells, which are thought to contribute to oxidative damage in neurons. Cryo-electron tomography has been used to study mitochondrial morphologies in photoreceptor synaptic termini (Perkins et al., 2012) and the Calyx of Held (Perkins et al., 2010), and protocols have been designed that would allow mitochondrial studies in axons as well (Shahmoradian et al., 2014). In addition, previous tomographic studies that measured neuronal membrane systems (Nickell et al., 2007; Gilliam et al., 2012) and intracellular transport systems (Gilliam et al., 2012) demonstrate the feasibility of using these approaches to investigate mitochondria as well. Future cryo-ET investigations correlating mitochondrial size and cristae surface area (a function of ATP synthesis levels) and the statistical analysis of axonal vesicles numbers could provide critical clues to understanding how defects in axonal transport promote dendritic spine loss and neurodegeneration.

The normal cycle of eukaryotic biosynthetic processes includes lysosomal degradation of lipid membranes and proteins. Abnormal accumulation of this cellular debris lies behind disease progression in lysosomal storage disorders and may play a similar role in other diseases associated with cellular accumulations. Protein misfolding and aggregation results in accumulations of α-synuclein found in Parkinson’s Disease (Lee et al., 2014) as well as accumulations of tau and amyloid-beta polypeptides responsible for the hallmark neuritic plaques and neurofibrillary tangles associated with Alzheimer’s Disease (Bloom, 2014). It is not presently clear whether synaptic peptide accumulations are strictly extracellular or whether accumulations occur within spines as well. However, synapses are armed with machinery for both exocytosis and endocytosis as it pertains to vesicle fusion, vesicle retrieval, and membrane recycling. Alterations in these processes within dendritic spines may lead to intraterminal protein accumulations that would be visible by cryo-ET. While few tomographic studies have focused on transgenic models of neurodegenerative disease (Frank et al., 2010; Gilliam et al., 2012), recent studies point to the value of cryo-ET as a critical tool for evaluating neurotoxic protein aggregation (Nicoll et al., 2013; Shahmoradian et al., 2013) as a means of evaluating such diseases. Future application of cryo-ET towards neurological dysfunction will likely reveal clues to understanding abnormal protein aggregation allowing that data to be integrated into models of disease pathogenesis.

All EM work and cryo-ET in particular still requires a great deal of manual intervention from human operators, despite great advances in analytical tools. Most large scale studies employ sparse reconstruction strategies whose resolution is limited by the sampling method employed in order to make data acquisition and analysis feasible (Harris et al., 2006). Dense reconstruction awaits improvements in the computational tools required to analyze the large data sets involved, including: (1) automatic alignment and segmentation of sections; and (2) generation of 3D reconstructions. While cryo-ET data appears to face some of the greatest hurdles in its potential integration into larger scale models of synaptic function and dysfunction, it also offers some of the greatest promise toward a larger understanding of mechanisms of signaling beyond simple connectivity.

## Computational tools for automated spine analysis

As current high-resolution imaging techniques allow for the collection of the massive amounts of 3D anatomical data or data series needed to feed systems level models, the bottleneck in these studies has become accurate quantification and classification of anatomical structures. Manual spine analysis is very time consuming and it is not feasible especially for the analysis of a large set of 3D and 4D images. Besides, the results are hard to reproduce due to investigator subjectivity and variability. Programs which can automatically and rapidly detect and measure spines of large datasets of 3D neuronal images with little human intervention are much needed to study the mechanisms regulating spine morphology. In this section we will first describe the general pipeline for automatic spine detection. Then, some existing popular tools for automated detection and quantitative analysis of spines will be presented, as well as their pros and cons. Major challenges and some new desired functions of the software will be briefly discussed at the end.

A general pipeline for spine detection is composed of image preprocessing, segmentation, spine labeling and postprocessing. The purpose of preprocessing is to increase the signal-to-noise ratio of the image so that it can be better segmented. Two most commonly used preprocessing techniques are denoising and deconvolution. Noise can be caused by the microscope or a detector such as a charge-coupled device (CCD) camera or photomultiplier tube (PMT). Low pass filters such as Gaussian filter or median filter are usually used to remove the speckle noise. Deconvolution is applied to revise the optical distortion that takes place during microscopic imaging. Depending on whether the point spread function (PSF) is known, there are two types of deconvolution methods available. Blind deconvolution is widely used in fluorescence microscopic image restoration which has two different approaches. The Richardson–Lucy deconvolution algorithm is the most commonly used iterative algorithm, while the Wiener deconvolution is the most common non-iterative algorithms for blind deconvolution.

The purpose of segmentation is to distinguish the foreground objects (e.g., dendrites and spines) from the background of the image. Many segmentation methods have been proposed for spine identification. The segmentation based on a global threshold can be easily applied (Koh et al., 2002); however, it is difficult to segment faint or thin spines without distorting the spine shapes with a single global threshold. Adaptive thresholding, which computes local thresholds, can partially solve the problem by calculating local thresholds at different regions (Cheng et al., 2007; Rodriguez et al., 2008). Many more sophisticated methods such as level set and Bayesian segmentation are also used (Fan et al., 2009; Oriented Markov Random Field Based Dendritic Spine Segmentation for Fluorescence Microscopy Images by Cheng). In many cases, these kinds of methods have superior segmentation results than the threshold based methods. However, the results can also be largely degraded because of the poor image quality, e.g., image with low SNR (signal to noise ratio).

To detect each single object, labeling is performed after segmentation. A major challenge toward automated spine detection and classification is laying out the criteria that define dendritic spines. This makes automated detection difficult to surpass manual detection. A common approach is based on the medial axis of dendrites which can be obtained by skeletonization. The spines are detected as the small protrusions along the boundaries of the dendrites. Usually diameter estimation is performed after skeletonization for dendritic morphometry. One simple way is to assume that the branch cross-section at any node is approximated as circular, which, however, will introduce quantization errors for small structures such as thin dendrites and spines. Rayburst sampling algorithm has been proposed to solve the problem. It is capable of continuous and more accurate radius estimation by using the original grayscale data instead of the segmented images. (Rodriguez et al., 2006).

After labeling, postprocessing is performed to refine the final results. For example, to separate two attached spines, or to remove the false spines, the detection of which can be caused by nearby axon pieces. If necessary, manual editing is also performed at this stage.

**Figure 4:**
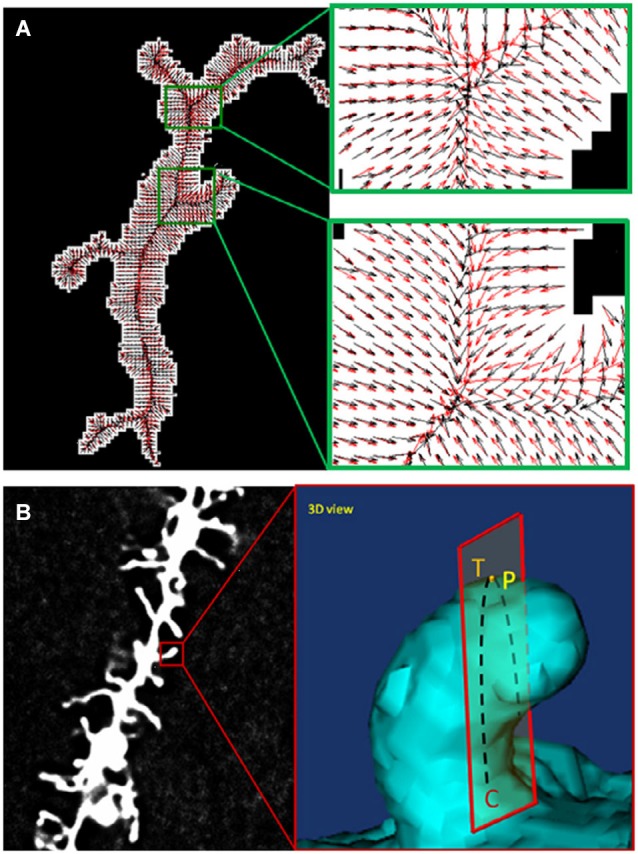
**Comparison of two alternative spine detection algorithms**. **(A)** Gradient vector field (GVF) analyses for centerline extraction (reprinted from Zhang et al., 2010). **(B)** Detection of Spine Tip Area Using Minimal Cross-Sectional Curvature (reprinted from He et al., 2012).

During the past decade, several software tools have been developed for automatic dendritic spine analysis. NeuronIQ was developed by Dr. Stephen Wong’s lab (Figure 4) with two versions designed for 2D and 3D analysis respectively (Cheng et al., 2007; Zhang et al., 2007, 2010). The 2D version is based on the maximal intensity projection (MIP) of image stacks and has much less computational complexity compared with the 3D versions. The 3D version provides 3D rendering and visualization. For both versions, measurement results can be exported to files using standard formats for further processing, modeling and statistical analysis. NeuronStudio was developed by Rodriguez et al. (2006) for automatic tracing and reconstruction of neuron structures from confocal image stacks. Rendering and 3D visualization are also provided. Imaris is a commercial software which can be used for dendritic spine analysis (Swanger et al., 2011). The Filament Tracer module provides the functioning of automatic spine detection and is capable of processing a large dataset of time-lapse images. Neurolucida is a powerful tool for neuron reconstructions and quantitative analysis from microscopy images (Aguiar et al., 2013). AutoSpine can be used for automatic detection and quantitication of dendritic spines based on the tracing results from Neurolucida. Some popular bioimage informatics platforms such as ImageJ have also been used for automated spine analysis. For example, Orlowski and Bjarkam (2012) proposed a simple method of spine counting based on the binarization and skeletonization functions provided by ImageJ.

All of the above mentioned software tools are capable of the fast, accurate, and automated quantification of dendritic spines, however with relative strengths and weaknesses. NeuronIQ and NeuronStudio are in house developed tools which are specially designed for dendritic spine analysis. They are standalone tools which are the easiest to be used nevertheless with the least flexibility, i.e., it is hard for the users to check the codes and introduce some new functionality by themselves. FilamentTracer and AutoSpine are modules of another software package and cannot be used alone. For example, FilamentTracer depends on Imaris for visualization, analysis and segmentation; while AutoSpine analyze the dendritic spines based on the images of dendritic branches obtained from Neurolucida and AutoNeuron. No doubt it is more complex for the users. However, with the support of other modules, FilamentTracer and AutoSpine are more powerful in visualization and data analysis. ImageJ has the highest flexibility and is the most powerful tool for users with some programming knowledge. The users can design their own approaches for dendritic spine analysis, with the help of the ever increasing plugins pool. The users can also change the plugins by themselves according to some specific requirements of the projects, since all the plugins’ source codes are publicly available.

One of the major challenges still faced for automated spine detection is the poor performance when processing images with complex backgrounds (e.g., with complex patterns of crossing neuronal projections) or when spine density is very high. Another challenge is to deal with the partial volume effects or a large spacing between neighboring slices. Additionally, the existing tools only focus on the structural and morphological analysis of dendritic spines. For a comprehensive integration of the many varied types of spine imaging data available, it is necessary to develop methods for analysis and incorporation of spine functional data. For example, to include trafficking of specific macromolecules into spines, or correlate the trafficking of synaptic molecules with morphological parameters by investigating the fluorescence intensity of dendritic spine heads. Additionally, the aforementioned tools are designed to process data sets of images of high contrast labeled neurons. While signal to noise issues in these samples is not trivial, it pales in comparison to the issues faced when attempting to analyze data from the label-free imaging modalities highlighted earlier. The generation of a whole new arsenal of new computational tools to process, analyze, and quantify these types of imaging data forms a major obstacle to their potential integration into models for the prediction of neuronal function and dysfunction.

## Tying it all together: correlations between changes in cell shape and changes in gene networks

**Figure 5:**
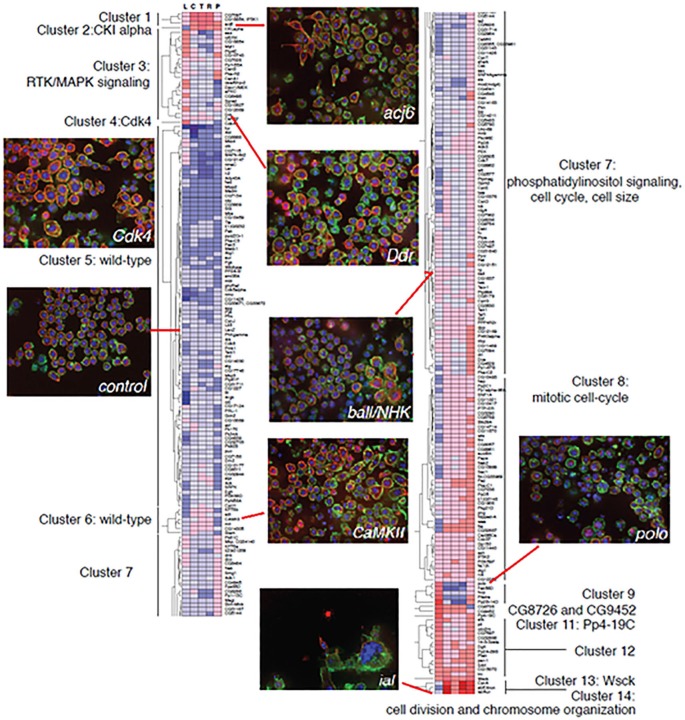
**Example of raw cell morphology imaging data of cells with gene-specific knockdowns and the corresponding gene expression profiling**. Data gathered reveals clusters of co-expressed genes that correlate with transitions to specific morphological states.

A prototype for incorporating anatomical changes into systems level models of cell biology activity serves as a blueprint for our third step, the integration of analyzed neuronal imaging data into a comprehensive systems model of brain function. Recently, Yin et al., building on previous work by Bakal et al., employed one of the first systems level approaches to correlating cell morphological complexity to gene function (Bakal et al., 2007; Yin et al., 2013, 2014). After light microscopic imaging of populations of individual cells in culture, *Drosophila* KC-167 cells were classified into five distinct groups based on morphology. A Quantitative Morphological Signature (QMS) was established and used to quantify cell shape changes by comparing to each of these groups. Gene signaling networks were then altered using RNAi (Figure 5) and the effects of these gene network alterations on population complexity properties, including cell number and distribution of cell shapes within a population, were analyzed. Work was performed in insect cells as well as mouse and human metastatic melanoma cells and showed that cells exhibit discrete changes in morphology correlating to low energy regions in shape space in response to changes in gene function. Based on the morphological profile generated by their alteration, genes then separate themselves into groups. This work showed that classes of genes conserved across species induce similar changes in cell morphology and that many of these mechanisms are likely conserved among cell types. This suggests that gene networks have evolved to tightly control the topology of a cell’s space shape in response to external and internal stimuli.

Previous work has successfully used this approach on cells which adopt a neuron-like morphology (Bakal et al., 2007) and despite the increasing levels of complexity involved, this concept likely applies to the morphology of adult neurons and their individual subcellular structures in the context of normal and pathological stimuli, allowing the correlation of genetic, developmental, and environmental changes to alterations in the balance of populations of structures. Dendritic spines, in particular provide an excellent substrate into which to expand this approach due to their abundance and well-documented variation in size, shape, and location. Investigators attempting to perform similar studies on neurons are faced with variations in the synaptic inputs received as well as neuronal subtype heterogeneity. Still, the major hurdle in realizing neuronal morphology correlation with gene and -omic data on a systems level is the need for population-level automated analysis of dendritic spines.

## Discussion

As the days of studying individual biological processes on the level of single entities operating in a vacuum give way to a more comprehensive approach that acknowledges the complex interplay between the many participants in a biological system, new data that can enrich systems models are at a premium. The correlation between the anatomy and function of neurons is well documented and many changes to normal neuronal anatomy are known to occur in various neurological disease states. One of the most prominent anatomical features of excitatory and other types of neurons, dendritic spines, which are the postsynaptic terminus of glutamatergic neurons, have long served as one of the most common anatomical indicators of neuronal function and dysfunction, including development, synaptic plasticity, and aging as well as schizophrenia, epilepsy, and Alzheimer’s disease. While helpful in directed spine counting experiments aimed at exploring the mechanism and severity of diseases, the large-scale, systematic imaging data of populations of neurons forms a largely untapped resource for integration with other data sources, such as proteomics or gene expression profiling, into comprehensive, systems medicine models of brain function and dysfunction. The old adage that a picture is worth a thousand words certainly applies in this case, as the potential imaging sets involved hold huge potential as big data sources. The exploitation of that resource faces many non-trivial obstacles in the acquisition, analysis, and modeling of imaging data, which we point out in this review.

Advances in microscopy technologies coupled with the exploding field of genetically encoded indicator proteins and their directed expression has given investigators the tools to gather very specific, high resolution imaging data in a number of contexts including in live, awake animals. The principle hurdle in incorporating such data sets into systems models is the development of algorithms that can convert large amounts of irregular imaging data into categorized quantitative data appropriate for those models. Large strides have been made in the development of automated or semi-automated software tools that detect and characterize dendritic spines from labeled neurons, but work does still remain in order to achieve accurate, fully automated spine detection, particularly from high density or low signal-to-noise images. These shortcomings are greatly magnified when dealing with more complex, yet more information rich images such as those attained from label-free imaging. While it is unlikely that any one size fits all solution exists, constant improvement in the accuracy of automated dendritic spine detection and characterization from images acquired from a variety of imaging modalities is already underway. Label-free multi-modal imaging of specific aspects of the surrounding tissues offers an excellent prospective method to place high resolution dendritic spine imaging in the proper biological context. The integration of characterized dendritic spine imaging with multimodal imaging of the surrounding environment and the wealth of currently available omics and brain functional data is the next step in the evolution of modeling brain function and dysfunction.

## Conflict of interest statement

The authors declare that the research was conducted in the absence of any commercial or financial relationships that could be construed as a potential conflict of interest.
